# Increase in peak oxygen uptake and Andersen test performance in children from age six to ten: The Health Oriented Pedagogical Project (HOPP)

**DOI:** 10.3389/fphys.2022.976505

**Published:** 2022-09-29

**Authors:** Asgeir Mamen, Morten Lindberg, Per Morten Fredriksen

**Affiliations:** ^1^ Kristiania University College, School of Health Sciences, Oslo, Norway; ^2^ Central Laboratory, Vestfold Hospital Trust, Tønsberg, Norway; ^3^ Faculty of Health and Social Sciences, Inland Norway University of Applied Sciences, Lillehammer, Norway

**Keywords:** longitudinal, cross-sectional, oxygen uptake, children, running, Andersen test longitudinal

## Abstract

The increased prevalence of non-communicable disease risk factors among children because of lack of physical activity is concerning. The Health Oriented Pedagogical Project was set up to combine learning activities and physical activity, thus reducing sedentariness during school time. The current study aimed to measure and describe the longitudinal and cross-sectional development of oxygen uptake and running performance in children at ages six and ten. The validity of the Andersen Test in predicting V̇O_2peak_ in these age groups was also evaluated. Eighty-six children (53 boys, 33 girls) with complete datasets at ages 6 and 10 years were included in the longitudinal study, while 192 children (106 boys, 86 girls) were included in the cross-sectional analysis because they missed data from 1 year. Oxygen uptake was measured using a metabolic analyser and maximal treadmill running, while the distance covered during the AT determined running performance. Body mass, height, and waist-to-height ratios were recorded. Multiple regression analysis was used to assess the association between oxygen uptake and running performance. The cross-sectional results did not differ from the longitudinal data for anthropometrical data, oxygen uptake and running performance. Height, body mass and waist-to-height ratio did not differ between the sexes at ages six or ten. Boys had significantly higher peak oxygen uptake than girls at 6 years of age, irrespective of how oxygen uptake was expressed. Allometric scaling of oxygen uptake revealed differences between sexes at both ages. Longitudinal running performance increased in both sexes from 6 to 10 years. Boys ran significantly longer only at age ten. The association between oxygen uptake and running performance varied according to how the oxygen uptake was expressed and with sex and age. Ten-year-old girls had the highest correlations in the longitudinal investigation, from *r* ^2^ = 0.48 (fV̇O_2peak_) to 0.65 (rV̇O_2peak_) between AT and V̇O_2peak_. The AT was found to be as valid as the 20-m shuttle run test in estimating peak oxygen uptake, with a random measurement error of approximately 11% of mean values.

## 1 Introduction

As high aerobic fitness is associated with a reduced risk of developing non-communicable diseases (Anderssen et al., 2007), measuring aerobic fitness has become a focus of public health studies and is performed by recording peak oxygen uptake (V̇O_2peak_). When testing large groups, directly measured oxygen uptake is often impractical due to time and cost constraints, soliciting the need to estimate V̇O_2peak_ using indirect tests. One test, the 20-m shuttle run test (20 mSRT) developed by Léger and Lambert (1982), has been extensively used and has been evaluated for validity and reliability with equivocal results (Mayorga-Vega et al., 2015; Armstrong and Welsman, 2019d). A more recent test developed for children, the Andersen test (AT), proposed by Andersen et al. (2008), is a 10-min, intermittent 20-m shuttle run test with 15 s work/rest intervals. Few studies have reported regression equations for predicting oxygen uptake relative to body mass and age using the AT (Andersen et al., 2008; Ahler et al., 2012; Aadland et al., 2014, 2018). The limits of agreement seem to be of the same order as that reported for the 20 mSRT (Liu et al., 1992; Melo et al., 2011; Aadland et al., 2018), but this should be verified with further studies. When studying the development of V̇O_2peak_, longitudinal data have several advantages over repeated cross-sectional data (Armstrong and Welsman, 2019c). Longitudinal data allow for the measurement of within-sample change over time, enable the measurement of the duration of events, and record the timing of various events (What Are Longitudinal Data? | National Longitudinal Surveys). Random variation in participants may skew the secular data collection to a larger degree and make interpretations of development of V̇O_2peak_ less exact.

In our study, we aimed to describe the longitudinal development of directly measured peak oxygen uptake using a treadmill test and the development of running performance in children aged 6 and 10 years using the AT. Further, we presented data for peak oxygen uptake and AT distance covered. Also, AT regression analysis for predicting V̇O_2peak_ at age 10 is presented.

## 2 Materials and methods

### 2.1 Participants

Overall, 270 children from the southeast region of Norway, born in 2008 and who attended nine different schools, were invited to participate in this study. In the first year, 166 students performed a valid peak oxygen uptake test. They had parental support on this occasion. Further, 138 of them had a valid AT, measured during school time. The baseline data were obtained in 2015 when the children reached the age of 6 years (first grade) and were tested annually up to 2020. Here data from ages 6 and 10 (fourth grade) is presented. The longitudinal study included the 86 children with valid peak oxygen uptake results and AT results for both 2015 and 2019 (53 boys and 33 girls). The cross-sectional data included all children with available valid data (V̇O_2peak_ and AT) from either 2015 or 2019 (106 boys and 90 girls).

### 2.2 Anthropometric measurements

Participant height was measured using a Seca 213 stadiometer (SECA GmbH, Hamburg, Germany) to the nearest 0.5 cm. Body mass was recorded to the nearest 0.1 kg, using a Tanita MC-980MA bioelectrical impedance assessment (BIA) scale (Tanita Corporation, Tokyo, Japan), with the participants dressed in light clothing, without shoes and socks. To compensate for clothing, 0.4 kg was subtracted from the measured value. Waist circumference was measured to the nearest 0.5 cm, according to the World Health Organization guidelines (WHO, 2008), with the participants standing erect and after a normal expiration. Body fat was estimated using the BIA scale and used for calculation of fat-free mass. The waist-to-height ratio (WHtR) was calculated by dividing the waist circumference (cm) by the height (cm) (Ashwell et al., 2012).

### 2.3 Aerobic fitness

The V̇O_2peak_ was recorded by direct measurements during maximal treadmill running. The treadmill used was a Matrix Ultimate Deck T-3X-04-F (Matrix Fitness, Cottage Grove, WI, United States). The choice of treadmill protocol can influence the test results; if the increments between each step are too large, the test may end prematurely (Fredriksen et al., 1998). Initially, they were allowed to walk on the treadmill at self-selected speeds for approximately 5 min, which was extended up to 15 min, if necessary. When they could run comfortably on the treadmill, testing was initiated using the protocol of Resaland (Resaland et al., 2009). The starting speed was 1.39 m·s^−1^ (5 km·h^−1^) at a 2.8° inclination (5%). Speed was maintained for 5 min and served as a warm-up. With the inclination remaining constant, the speed was increased to 1.94 m·s^−1^ (7 km·h^−1^) and subsequently increased by 0.28 m·s^−1^ (1 km·h^−1^) each min until a speed of 2.78 m·s^−1^ (10 km·h^−1^) was attained. From this point, a constant speed was maintained while the inclination was increased by 0.57° (1%) every minute until the test ended.

For safety reasons, the test leader stood behind each participant on the treadmill and held onto them by a backpack belonging to the metabolic analyser. The analyser itself was placed on the treadmill. Parental support was provided during the test for the 6-year-old participants. Verbal encouragement was provided at the end of the test. If the child wanted to stop at any point, they grasped the handrail of the treadmill, and the test leader stopped the treadmill. A test was considered valid if at least one of the following criteria were satisfied: a plateau in oxygen uptake was evident, the Respiratory Exchange Ratio (RER) was higher than 0.98, the heart rate was more than 198 (the median of the HR_max_), or the test supervisor judged the effort to be maximal. A plateau was recorded when the oxygen uptake rose less than 2.5 ml·kg^−1^·min^−1^ from one stage to another. The median value for the highest three consecutive 10 s measurements at the end of the test was recorded as the peak value. The treadmill was calibrated for inclination and speed at the start of every test cycle.

At the age of 6 years, a Cosmed K4b2 metabolic analyser in breath-by-breath mode was used to measure oxygen uptake; later, a Cosmed K5 metabolic analyser (Cosmed Srl, Rome, Italy) with a micro mixing chamber was used. The accuracy of these analysers (K4b^2^, K5) is well documented (Duffield et al., 2004; Perez-Suarez et al., 2018; Crouter et al., 2019; Ross et al., 2020). The children wore Hans Rudolph 7400 V2 face masks (Hans Rudolph Inc., Shawnee, KS, United States) in “petit” size for the tests.

Scaling of oxygen uptake relative to body mass has been extensively used since the seminal study by Robinson (1938). Bergh et al. found an exponent closer to 0.75 (1991) to be the preferable choice. Conversely, Pettersen et al. (2001) advocated the use of scaling body mass to 0.67, based on the development of oxygen uptake and running performance. Armstrong and Welsman ((2019b) suggested using fat-free mass when describing the development of children’s oxygen uptake. Lolli et al. found no difference between boys and girls with a fat-free body mass exponent of 0.90 (2017).

Therefore, the measured oxygen uptake was expressed in four ways: absolute form (ml·min^−1^), relative to body mass (ml·kg^−1^·min^−1^), allometrically scaled with body mass raised to the power of 0.67 (ml·kg^−0.67^·min^−1^), and relative to fat-free mass (ml·_FFM_kg^−1^·min^−1^).

Heart rate (HR) was recorded using a Polar H3 (Polar Electro OY, Kempele, Finland) sender that sent signals to a Polar RS100 HR monitor, the Polar HR receiver unit on the metabolic unit, and the treadmill display.

The AT was performed as described by Andersen et al. (2008), as an intermittent 20-m shuttle run, with 15-s work/rest intervals for 10 min. During the running phase, music was played; when the music stopped, the children were instructed to halt too. Before the test, they were instructed to maintain an even pace and run as quickly as possible. To compensate for the stopping distance, they were asked to back up a few steps at each stop so that the actual running distance could be recorded. The test personnel recorded the number of laps completed by the child during the test. The number of laps was converted to distance in metres (number of laps × 40), and the completed portion of the last lap was added to obtain the total distance. Tests were invalidated if the participant did not run the whole length of the marked distance (20 m), stopped before 10 min had passed, or ran with an uneven pace (mostly walking, but with occasional sprints).

### 2.4 Ethics

The Regional Committee approved the study protocol for Medical Research Ethics and the Norwegian Social Science Data Service (2014/2064/REK South-East). This study was conducted in accordance with the tenets of the 2013 Declaration of Helsinki. Parents/guardians of the children provided written informed consent to the project leader before data collection. The study was retrospectively registered as a clinical trial (ClinicalTrials.gov Identifier: NCT02495714) as of 20 June 2015. Baseline values were collected from mid-January 2015.

### 2.5 Statistical analysis

Results are presented as mean ± standard deviation (SD) unless otherwise noted. Differences between the sexes and ages were analysed using the Gosset’s (Student’s) independent *t*-test and Cohen’s *d*. Using Fisher’s transformation, differences between the correlation coefficients were analysed according to the method described by Lenhard and Lenhard (2014). Multiple regression was used to establish equations for predicting oxygen uptake with AT and BM as variables. The effect of body mass on oxygen uptake was investigated by regressing oxygen uptake with BM or FFM. To control for maturity, we used multiple regression with the independent variables AT, age, height, and BM.

Statistical analyses were performed using NCSS 2022 Statistical Software (NCSS LLC., East Kaysville, Utah, United States) and SigmaPlot 14.5 (Systat Software GmbH, Erkrath, Germany). Statistical significance was set at *p* < 0.05. However, this does not imply that there is no difference, or that a difference is unimportant if the *p*-value exceeds this arbitrary limit (Amrhein et al., 2019).

## 3 Results

### 3.1 Anthropometry

A description of the longitudinal anthropometric data of the children aged 6 and 10 years is presented in Table 1. From 6 to 10 years, there was a significant development of all anthropometric variables, except for the Waist to Height Ratio (WHtR), for both sexes. There was no significant difference between the sexes at 6 or 10 years of age.

**TABLE 1:** Longitudinal anthropometric data, results are mean (standard deviation). BM, body mass; FFM, fat-free mass; WC, waist circumference; WHtR, waist-to-height ratio. *p < 0.01, between 6 years and 10 years.

	Boys (*n* = 53)		Girls (*n* = 33)	
	6 years	10 years	6 years	10 years
Age (years)	6.72 (0.37)	9.64 (1.70)*	6.71 (0.31)	9.47 (2.13)*
Height (cm)	123.4 (5.2)	146.1 (6.1)*	122.3 (5.2)	145.4 (7.2)*
BM (kg)	23.8 (4.2)	39.0 (9.0)*	23.7 (3.6)	38.5 (7.6)*
FFM (kg)	19.4 (2.5)	30.2 (4.5)*	18.6 (2.5)	29.2 (4.8)*
WC (cm)	57.3 (5.0)	68.2 (9.7)*	57.3 (5.1)	66.9 (8.5)*
WtHR	0.46 (0.03)	0.47 (0.06)	0.47 (0.04)	0.46 (0.05)

### 3.2 Development of peak oxygen uptake and Andersen test

**FIGURE 1:**
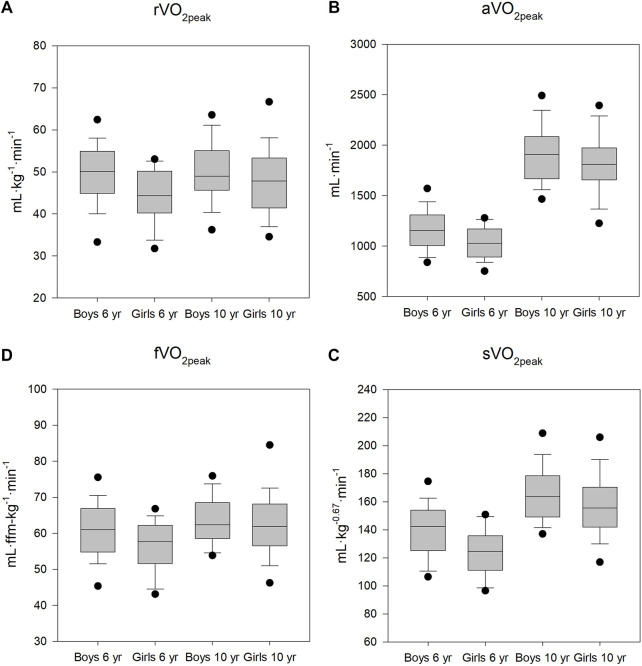
Peak oxygen uptake at ages 6 and 10. **(A)**: oxygen uptake relative to body mass. **(B)**: absolute oxygen uptake. **(C)**: oxygen uptake allometrically scaled (body mass ^−0.67^). **(D)**: oxygen uptake relative to fat-free mass. *significant differences between boys and girls (*p* < 0.01). Boxes are 25–75 percentiles, whiskers 5 and 95 percentiles. Horizontal line is median. Dots are outliers.

For longitudinal V̇O_2peak_, irrespective of how it was expressed, the boys had significantly higher values than the girls (*p* < 0.01) at the age of 6, but not at the age of 10. The development of oxygen uptake is shown in Figure 1, which presents the V̇O_2peak_ expressed in four ways: absolute, in ml·min^−1^ (aV̇O_2peak_); relative to body mass, in ml·kg^−1^·min^−1^ (rV̇O_2peak_); allometrically scaled to body mass raised to the 2/3 power, in ml·kg^−0.67^·min^−1^ (sV̇O_2peak_); and relative to fat-free mass, ml·fat-free mass kg^−1^·min^−1^ (fV̇O_2peak_). From 6 to 10 years, all peak oxygen uptake values increased significantly among the girls (*p* < 0.01). For boys, rVO_2peak_ did not increase significantly (*p* = 0.053), whereas the other peak oxygen uptake values did (*p* < 0.02). The percent changes from 6 to 10 years of age were as follows: rV̇O_2peak_: 1.5% for boys and 6.8% for girls, aV̇O_2peak_: 45.0% for boys and 42.0% for girls, sV̇O_2peak_: 20.0% for boys and 20.0% for girls, fV̇O_2peak_: 10.0% for boys and 13.8% for girls.

**FIGURE 2:**
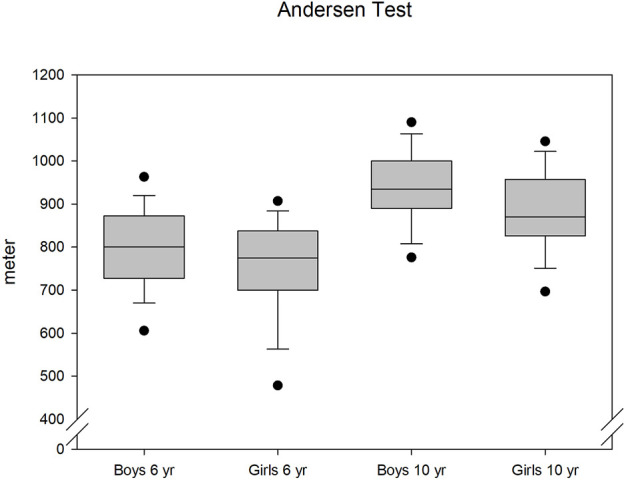
Development of running distance in the Andersen test.

Longitudinal running performance was significantly increased in both sexes from ages 6–10 years (*p* < 0.01). At the age of 6 years, there was no significant difference in AT performance between boys and girls (*p* = 0.09); however, at the age of 10 years, the difference was significant (*p* = 0.01), with the boys able to run longer than the girls. The running performance increased by approximately 18% for both sexes (boys: 148 m and girls 133 m) (Figure 2). A comparison of the results for each year (cross-sectional) with the longitudinal results revealed only minor, non-significant differences in oxygen uptake and running performance. Cohen’s *d* effect sizes between longitudinal and cross-sectional data ranged from 0.01 to 0.16 for the different ways of expressing oxygen uptake and AT.

#### 3.2.1 Change in work economy

The 5-min stage in the treadmill protocol was used both as a quality check of the oxygen uptake measurements, and to measure work economy. There were only insignificant differences between the boys and girls, and the work economy increased by approximately 20% from 6 to 10 years old. rV̇O_2_ was 33 ml·kg^−1^·min^−1^ in the 1st year and decreased to 26 ml·kg^−1^·min^−1^ in the final year. This is a reduction in energy expense of 1.75 ml·kg^−1^·min^−1^ pr., year.

### 3.3 Predicting peak oxygen uptake with Andersen test

Using the original equation for boys and girls suggested by Andersen et al. (2008), under-estimated the average rV̇O_2peak_ for 10-year-olds in this study with 5.9 ml·kg^−1^·min^−1^. The Ahler study, involving 9-year-olds, under-estimated rV̇O_2peak_ with 2.4 ml·kg^−1^·min^−1^. Only AT was included in these equations. The equations set out by Aadland et al. (Aadland et al., 2014, 2018) used BM in addition to AT. The 2014 equation (10-year-olds) over-estimated the rV̇O_2peak_ with 4.0 ml·kg^−1^·min^−1^. The 2017 equation (10-year-olds) also over-estimated the rV̇O_2peak_: 4.9 ml·kg^−1^·min^−1^.

With AT and BM as variables, the *R* ^2^ for the different ways of expressing oxygen uptake ranged from 0.559 (Boys, rV̇O_2peak_) to 0.271 (Girls, fV̇O_2peak_), The Mean Absolute Per centage Error (MAPE) was approximately 11%. See Table 2.

**TABLE 2:** Best regression equations at 10 years of age using AT and BM.

Boys			
Oxygen uptake		*R* ^2^	SEE
rV̇O_2peak_	34.988 + 0.037 × AT − 0.504 × BM	0.559	5.49
aV̇O_2peak_	−379.776 + 1.457 × AT+ 23.732 × BM	0.523	211.68
sV̇O_2peak_	9.319 + 0.125 × AT − 0.521 × BM	0.397	18.10
fV̇O_2peak_	37.814 + 0.037 × AT − 0.263 × BM	0.353	6.59
**Girls**			
**Oxygen uptake**		***R*** ^**2**^	**SEE**
rV̇O_2peak_	32.588 + 0.052 × AT − 0.420 × BM	0.462	6.69
aV̇O_2peak_	−483.136 + 1.398 × AT+ 27.128 × BM	0.459	252.10
sV̇O_2peak_	57.869 + 0.120 × AT − 0.174 × BM	0.325	21.98
fV̇O_2peak_	37.814 + 0.037 × AT − 0.263 × BM	0.271	8.46

AT, adersen test result; BM, body mass; SEE, standard error of estimate.

#### 3.3.1 Effect of body mass on oxygen uptake

Regressing the oxygen uptakes related to a form of BM with BM or FFM showed sV̇O2 and fV̇O2 to be affected. Using results from both age groups differed somewhat from using either first year, as 6-year-olds or last year, as 10-year-olds.

Using results from both 6-and 10-year-olds gave a non-significant slope for fV̇O_2_ (*p* = 0.205). The same was true for sV̇O2 in the only 6-year-olds (*p* = 0.777). Else, the differences between the various age groups were not statistically significant. Table 3 gives the details.

**TABLE 3:** Effect of controlling for BM or FFM.

	Variable	Correlation	*R* ^2^≤	Slope	Slope *p*≤	Sample
rV̇O_2_	BM	−0.205^1^	0.042	−0.172	0.001	Both 6-yr-age and 10-yr-age
sV̇O_2_	BM	−0.308	0.095	−0.856	0.001	
fV̇O_2_	FFM	0.071^2^	0.005	0.096	**0.205**	
rV̇O_2_	BM	−0.542	0.294	−0.600	0.001	Only 10-yr-age
sV̇O_2_	BM	−0.256	0.066	−0.818	0.001	
fV̇O_2_	FFM	−0.261	0.068	−0.513	0.001	
rV̇O_2_	BM	−0.393	0.154	−0.594	0.001	Only 6-yr-age
sV̇O_2_	BM	−0.023^1^	0.001	−0.093	**0.777**	
fV̇O_2_	FFM	−0.150	0.023	−0.456	0.071	

Significantly different from the two other age groups. ^2^Significantly different from the 10-yr group.

Bold types indicate a non-signifcant slope.

The regression equation that provided the best fit for predicting peak oxygen uptake varied between the sexes and in terms of how oxygen uptake was expressed. Only the data from the 10-year-olds were used to estimate oxygen uptake, as that gave the best fit. For the 6-year-old children, *R* ^2^ for rV̇O_2_ was 0.10 for boys and 0.18 for girls, while at the age of 10 years, the corresponding *R* ^2^ was 0.45 and 0.65. The same was true for the other expressions of oxygen uptake (not shown), and these differences between 6 and 10 years of age were significant (*p* < 0.05).

Using age, height, and BM as variables to control for maturity, MAPE error was reduced to approximately 9%. *R* ^2^ was highest with rV̇O_2peak_ (Girls rV̇O_2peak_) and lowest in fV̇O2_peak_ (Boys, fV̇O_2peak_).

## 4 Discussion

The principal findings of the present study were as follows: 1) we found no significant differences between the cross-sectional and longitudinal results for anthropometry and aerobic fitness; 2) when oxygen uptake was expressed relative to body mass, boys increased their oxygen uptake by 2% and their running performance by 18%, while girls improved their oxygen uptake by 7% with a similar increase in running performance as the boys; 3) the limits of agreement for the AT in estimating peak oxygen uptake was as those found for the 20 mSRT. Therefore, care should be exercised when commenting on individual results (Aadland et al., 2018).

### 4.1 Cross-sectional vs. longitudinal data

A few participants are inevitably lost to follow-up in longitudinal studies, reducing the overall sample size (N). This may introduce bias in the study. Our data indicated only minor differences. In addition, our data did not reveal any polarisation of results (where the best gets better, and the worst gets worse over time).

### 4.2 Development of peak oxygen uptake and running performance

An endurance running performance depends on several factors. Both the peak oxygen uptake, the ability to utilise the oxygen uptake (lactate or ventilatory threshold), the work economy and motivation for the task are crucial factors for performing well in running events (Åstrand and Rodahl, 1970; Bassett and Howley, 2000). Threshold results are of particular interest, as they combine both the “motor” (aerobic power) and the “economy” (utilisation of oxygen uptake and work economy). Both Mahon and Becker have investigated the ventilatory threshold in children, and found it a feasible variable (Becker and Vaccaro, 1983; Mahon and Marsh, 1993; Mahon and Cheatham, 2002). We have found it difficult to comment on ventilatory threshold data since we did not use the same analyser throughout the project.

Oxygen uptake can be expressed in several ways that offer a different perspective. Running performance, and other activities where BM is carried, may better be described with oxygen uptake scaled to the mass carried. There is a choice of using the whole BM, allometrically scaling it (raising BM to 2/3 or ¾ power) or using FFM, the BM without fat mass (Leam Body Mass). The conventional approach has been to scale oxygen uptake to BM, but this method has been critiqued by Armstrong and Welsman (Armstrong and Welsman, 2019a; 2019c). In this study we used both absolute oxygen uptake, oxygen uptake relative to BM, oxygen uptake relative to allometrically scaled BM (0.67) and oxygen uptake scaled to FFM as recommended by Loli et al. (2017) to present how the expression of oxygen uptake influences the relationship with running performance.

The running economy, as oxygen uptake relative to BM (ml·kg^−1^·min^−1^), improved significantly by 20%. This is probably because the technique for treadmill running improved during this period. Not more than five children had ever tried to run on a treadmill in the 1st year. The study of Cureton et al. (1997) found an improvement in treadmill running economy with a reduction of approx. 1 ml·kg^−1^·min^−1^·year^−1^ in children from 7 to 17 years of age. Speed was 8 km·h^−1^. We found a reduction of 1.75 ml·kg^−1^·min^−1^·year^−1^ from 6 to 10 years. One possible explanation is that the subjects in the present study initially exhibited a very high energy expenditure, as this was their first attempt to run on a treadmill. In the present study, the development of rV̇O_2peak_ did not follow the development of running performance. The distance covered in 10 min increased by 18% for boys (148 m) and girls (133 m); however, the rV̇O_2peak_ increased by only 2% for boys and 8% for girls. A better running economy may partly explain the difference in development of rV̇O_2peak_ and AT distance.

In Norway, a recent publication demonstrated that breast development (thelarche) begins at the age of 10.4 years, which is relatively earlier than previously considered (Bruserud et al., 2020). This age is somewhat higher than the age of the girls in our study (9.47 ± 2.13 years). Therefore, the improvement in V̇O_2peak_ relative to the body mass we observed in girls may be attributed to this lack of physiological development, as only small increases in fat deposits would have occurred up to this age.

At 6 years of age, we observed that oxygen uptake between girls and boys was significantly different, regardless of how it was expressed, although running performance was similar. At 10 years of age, oxygen uptake did not differ significantly between girls and boys; however, the overall running distance was significantly greater among boys. Therefore, girls showed a larger improvement in oxygen uptake than boys, albeit a lower gain in running performance (Figure 2). This highlights the fact that endurance performance is not entirely determined by the V̇O_2peak_.

Motivational factors represent the willingness to endure unpleasantness to reach a goal. This ability is steadily improved in children, and goal motivation is important for many children. In the first treadmill test, several children stopped running when they got tired but not exhausted. At age 10, their willingness to continue seemed to be higher, as they now often ended the test at the turn of a whole minute. During the AT running, most pupils could now run at a more even pace throughout the 10 min duration of the test.

Self-efficacy (the trust in one’s own skills to perform a job) is essential for physical activity and can affect personal performance. Cairney et al. (2008) observed that self-efficacy accounted for 9% of the total variation in shuttle run performance; children with higher self-efficacy ran longer in the shuttle run test. Thus, the boys might have had a better belief in their ability to perform and hence performed better than the girls at the age of 10, despite having almost equal V̇O_2peak_. Girls reach puberty before boys (Bruserud et al., 2020; Oehme et al., 2020) and at 10 years of age are usually more mature than boys. Maturation often leads to a shift in interests, going from physical activities to more sedentary ones, as levels of Moderate to Vigorous Physical Activity decrease with increasing age in children (Nader et al., 2008; Deng and Fredriksen, 2018). Therefore, boys may be more interested in physical activities at this age. In addition, it can be speculated that girls at this age are less competitive than boys. According to Niederle and Vesterlund (2011): Both laboratory and field studies largely confirm these initial findings, showing that gender differences in competitiveness tend to result from differences in overconfidence and in attitudes toward competition. If this holds true for children, it may partly explain our findings.

Not to be overlooked, physiological differences between the sexes can also affect the response to exercise. Armstrong et al. (1991) observed sex-related differences in heart rate response to exercise, possibly due to the higher haemoglobin (Hb) levels in boys. In their study, Fredriksen et al. (2018) demonstrated that Hb was an important variable influencing running performance in children.

In the study by Aadland et al. (2018), a significantly higher directly-measured V̇O_2peak_ was reported among 10-year-old boys compared to that in our study; however, the difference in running performance was not significantly different. This inconsistency may be due to differences in the AT protocols used. Aadland et al. (ibid.) defined a standard technique for turning 180° with one hand touching the floor for each turn and controlled it during the test. Our results are inflated as we did not control the turnings in such a way.

### 4.3 Validity of Andersen test compared with other shuttle run tests

One aim of our study was to determine the usefulness of AT in predicting V̇O_2peak_. There are several indirect tests for estimating V̇O_2peak_ based on performance (Binkhorst et al., 1986; Grant et al., 1995; Mayorga-Vega et al., 2016). The well-known 20 mSRT by Léger and Lambert (1982) has been extensively evaluated and shown to be valid and reliable (Mayorga-Vega et al., 2015). However, important uncertainties have been reported with the results obtained using this test (Armstrong and Welsman, 2019a). Mayorga-Vega et al. (2015) observed a moderate-to-high correlation coefficient for predicting oxygen uptake using this test, while Welsman and Armstrong (2019b) observed that the test was unsuitable for use in children aged 11–14 years Ruiz et al. (2009) concluded that the 20 mSRT was questionable for use at the individual level.

The uncertainty and error in predicting peak oxygen uptake with the use of AT have been reported in several investigations, with values close to what we have found (Table 4) (Andersen et al., 2008; Ahler et al., 2012; Aadland et al., 2018).

**TABLE 4:** Regression equations with control of maturity (age, height, and BM).

Boys													
	Constant	*p*≤	AT	*p*≤	Age	*p*≤	Height	*p*≤	BM	*p*≤	*R* ^2^	SEE	MAPE
aV̇O_2peak_	−1,359.171	0.14	+0.969	0.01	+22.106	0.22	+10.929	0.15	+18.434	<0.01	0.529	209.92	8.6
rV̇O_2peak_	+17.277	0.47	+0.024	0.01	+0.945	0.05	+0.162	0.36	−0.566	<0.01	0.539	5.52	8.2
sV̇O_2peak_	−0.911	0.99	+0.081	0.01	+2.622	0.10	+0.672	0.25	−0.839	0.04	0.293	18.19	8.3
fV̇O_2peak_	+63.526	0.03	+0.021	<0.05	+1.059	0.06	-0.174	0.41	−0.111	0.45	0.232	6.58	7.7
**Girls**													
	**Constant**	***p*** **≤**	**AT**	***p*** **≤**	**Age**	***p*** **≤**	**Height**	***p*** **≤**	**BM**	***p*** **≤**	***R*** ^**2**^	**SEE**	**MAPE**
aV̇O_2peak_	−2,369.533	0.01	+1.367	<0.01	−4.046	0.82	+17.776	0.02	+11.017	0.12	0.608	206.56	8.7
rV̇O_2peak_	−18.136	0.45	+0.037	<0.01	−0.462	0.76	+0.483	0.02	−0.925	<0.01	0.656	5.52	8.7
sV̇O_2peak_	−108.865	0.17	+0.122	<0.01	−0.505	0.74	+1.586	0.02	−1.723	0.01	0.522	18.14	8.6
fV̇O_2peak_	+6.721	0.4	+0.05	0.01	+0.122	0.85	+0.265	0.33	−0.63	0.02	0.434	7.69	9.6

For oxygen uptake (V̇O_2peak_), prefixes a, r, s, and f represent absolute, relative to body mass, allometric scaled body mass and relative to fat-free body mass, respectively. SEE, is Standard Error of Estimate, and MAPE, is mean per cent error of forecast.

Andersen et al. (2008) did not detect a significant difference between two test runs (difference of 15 m, *p* = 0.10). Aadland et al. (2014) observed a learning effect in AT performance and recommended performing two tests to obtain valid results. Practising a test before a maximal performance may be helpful. A learning effect is also probable regarding maximum-effort exercise on a treadmill, especially when wearing a face mask.

From birth to late childhood, children grow in average height from 0.5 to 1.5 m, and their average body mass increases from 3.5 to 35 kg. Thus, in growing individuals, the way oxygen uptake is expressed is important for obtaining a realistic picture of V̇O_2peak_ development (Welsman and Armstrong, 2019a; Armstrong and Welsman, 2019b). Relative to body mass, oxygen uptake during childhood is stable at about 50 ml·kg^−1^·min^−1^. Girls have somewhat lower values up to puberty (12–13 years), after which peak oxygen uptake is reduced relative to body mass due to increased body fat content, whereas the value for boys continues to improve. When oxygen uptake is expressed absolutely as ml (or L)·min^−1^, oxygen uptake increases steadily from approximately1000 ml·min^−1^ at the age of 5 years–3,500 ml·min^−1^ at the age of 15 years, with lower values among female participants (Åstrand, 1952). The use of allometric scaling has been suggested, particularly as aerobic power is believed to be correlated with performance (Pettersen et al., 2001). The amount of body fat is known to influence running performance (Armstrong and Welsman, 2019b); therefore, oxygen uptake relative to fat-free mass is also suggested as a suitable way of expressing aerobic power (Armstrong and Welsman, 2019b).

We observed both sV̇O_2 peak_ and fV̇O_2peak_ to be successful variables in nullifying the effect of body mass as the slopes were not significantly different from 0. This is in accordance with the recommendations of Armstrong and Welsman (2019c). Scaling oxygen uptake relative to body mass per se would reward lightweight and penalise heavier individuals. It should be emphasised that our fat measurement is based on Bioelectrical Impedance Assessment (BIA) measurements. BIA has gained acceptance for use in healthy populations, but sources of error can make the results erroneous. Therefore, measurements should be strictly standardised, which was not applied to our data collection. Talma et al. found BIA to be practical for measuring children but unable to accurately assess per cent body fat, fat mass or FFM (2013). In their systematic review, Chula de Castro et al. found a high correlation with reference methods but an underestimation of body fat (2018).

According to Tønnessen et al. (2015), the performance development of the Norwegian elite track and field athletes aged 11–18 years is similar for boys and girls up to 12. Using a 9-min running test, Golle et al. (2015) demonstrated that girls improved more between ages 9 and 12 than boys, although girls’ performance was lower at all ages. This test is similar to the AT in duration (9 and 10 min) and self-selected speed; however, it differs in that the AT is a shuttle run test. Heavier participants may be penalised, as there are several 180° changes in the running direction in the AT, forcing the participant to accelerate frequently. The difference in responses between the study by Golle et al. (ibid.) and our study may have been due to the inclusion of older children in the preceding study. Milanese et al. (2020) showed that sex influenced approximately 10% of performance in a 30-m dash in children aged 6–12 years. This test measured anaerobic capacity and showed equal development between the sexes in children aged 6 to 12. Age governed approximately 45% of performance.

In the present study, the best association between oxygen uptake and AT performance was observed when using rV̇O_2peak_ and body mass together with the distance covered in the AT. The correlation between AT and V̇O_2peak_ was significantly higher in 10-year-old children. In line with our findings, Aadland et al. (2018) showed a correlation in the improvement from 10 to 16 years of age. In the initial study by Andersen et al. (2008), a higher correlation was observed among participants aged 20–27 years than those aged 10 to 11. This finding suggests that increasing maturity improves the association between the two maximal exertion tests. This is expected as the motivation to sustain discomfort at maximal physical effort improves with maturity, and the ability to plan the effort increases with experience. When 6-year-old children performed the AT, it was common to see them run at full speed in the beginning, get exhausted within a few minutes, and then perform a final sprint on the last lap. Such pacing weakens the association between AT and aerobic power. The improved ability to control the initial pace at an older age may thus be reflected in the stronger association between AT and V̇O_2peak_ at the age of 10 years.

In the V̇O_2peak_ test, maintaining a high level of effort is challenging; hence, there may be a learning effect that can either improve or reduce performance. The next test may be less than maximal depending on whether the first experience was pleasant or unpleasant (Ekkekakis et al., 2011). Some children aged 10 years had a keen interest in their classmates’ performance and targeted a goal for their treadmill running. This was evident from the time to exhaustion, as the tests mostly ended at a full minute (i.e., 8.00, 12.00) in the 10-year-olds.

The face mask may have limited the results of the V̇O_2peak_ test. Even if a face mask is usually preferred over a mouthpiece, it can still be scary and unpleasant for a child, especially at a young age. Moreover, as the face mask makes breathing heavier, some children might have ended their treadmill test prematurely because of the discomfort and not the physiological fatigue. This unpleasantness might have affected the subsequent tests.

The considerable improvement in the technical ability to perform treadmill running, better pacing strategy in the AT, and a higher performance motivation indicates that maximal endurance testing for larger groups of children should be postponed until the age of approximately 10 years. Although some individuals can exert themselves maximally from a very young age, this does not apply to the average child.

### 4.4 Limitations of the study

The motivation and ability to perform the tests varied considerably between participants and across different ages. This may have weakened the findings and obscured the associations. The study was conducted over several years, and the data collection was performed by different people each year. Nine schools participated, and they all differed in floor surface for the AT. The testing at the various schools was performed at approximately the same time every year; however, the time of day for a particular participant was not standardised. Even if testing was conducted indoors, the outdoor temperature may influence the indoor temperature and change the test conditions.

### 4.5 Conclusion

The cross-sectional and longitudinal results showed no significant differences in aerobic fitness and running performance. The improvement of oxygen uptake and running performance deviated from each other. For the girls, the improvement of peak oxygen uptake was approximately half of the improvement in running distance when oxygen uptake was expressed relative to body mass (7% vs. 18%). The allometrically scaled oxygen uptake and fat-free mass uptake had improvements closer to the improvement in the running performance (20% and 14% vs. 18%). For boys, the relation between V̇O_2peak_ development and running length development was very poor, and oxygen uptake improved by only 2%, whereas the running performance increased by 15% when using rV̇O_2peak_. As with the girls, expressing the oxygen uptake absolutely or allometrically scaled improved the relationship between the two developments. The regression equations for the 6-year-olds gave a significantly poorer fit than those for the 10-year-old children. This can indicate that testing children below age 6 in maximal running tests may be questionable. AT was found to be valid for estimation of oxygen uptake. The accuracy of the test is on the level with the 20 mSRT. This implies that caution should be adopted when drawing conclusions about individual results but that the test works acceptably with group data.

## Data Availability

The raw data supporting the conclusion of this article will be made available by the authors, without undue reservation.
